# Indoor Positioning Based on Bluetooth Low-Energy Beacons Adopting Graph Optimization

**DOI:** 10.3390/s18113736

**Published:** 2018-11-02

**Authors:** Zheng Zuo, Liang Liu, Lei Zhang, Yong Fang

**Affiliations:** 1 College of Electronics and Information Engineering, Sichuan University, Chengdu 610064, China; b110434@stu.scu.edu.cn; 2 College of Cybersecurity, Sichuan University, Chengdu 610064, China; zhanglei2018@scu.edu.cn (L.Z.); yfang@scu.edu.cn (Y.F.)

**Keywords:** BLE-based indoor positioning, fingerprinting, graph optimization

## Abstract

Bluetooth Low-Energy (BLE) beacons-based indoor positioning is a promising method for indoor positioning, especially in applications of position-based services (PbS). It has low deployment cost and it is suitable for a wide range of mobile devices. Existing BLE beacon-based positioning methods can be categorized as range-based methods and fingerprinting-based methods. For range-based methods, the positions of the beacons should be known before positioning. For fingerprinting-based methods, a pre-requisite is the reference fingerprinting map (RFM). Many existing methods focus on how to perform the positioning assuming the beacon positions or RFM are known. However, in practical applications, determining the beacon positions or RFM in the indoor environment is normally a difficult task. This paper proposed an efficient and graph optimization-based way for estimating the beacon positions and the RFM, which combines the range-based method and the fingerprinting-based method. The method exists without need for any dedicated surveying instruments. A user equipped with a BLE-enabled mobile device walks in the region collecting inertial readings and BLE received signal strength indication (RSSI) readings. The inertial measurements are processed through the pedestrian dead reckoning (PDR) method to generate the constraints at adjacent poses. In addition, the BLE fingerprints are adopted to generate constraints between poses (with similar fingerprints) and the RSSIs are adopted to generate distance constraints between the poses and the beacon positions (according to a pre-defined path-loss model). The constraints are then adopted to form a cost function with a least square structure. By minimizing the cost function, the optimal user poses at different times and the beacon positions are estimated. In addition, the RFM can be generated through the pose estimations. Experiments are carried out, which validates that the proposed method for estimating the pre-requisites (including beacon positions and the RFM). These estimated pre-requisites are of sufficient quality for both range-based and fingerprinting-based positioning.

## 1. Introduction

Location has become increasingly important for many position-based services (PbS). However, although the global navigation satellite system (GNSS) can satisfy the needs for most outdoor situations, indoor positioning still remains a challenge. The abundant sensors embedded in today’s mobile devices have greatly enhanced their ability to sense the indoor environment, thus providing possibilities for indoor positioning. Many indoor positioning methods rely on pre-installed infrastructures and can provide reasonable accuracy, such as UWB ranging anchor-based [1,2], Wi-Fi access points (APs)-based [3,4], Ultrasound [5,6]-based and so on. Among these methods, Bluetooth low-energy (BLE) beacons have great potential due to their advantages:

- The positioning process is independent of extra hardware. After the BLE beacons have been installed, the only equipment needed is a BLE-enabled smart phone, while foot-mounted or waist-mounted inertial pedestrian positioning [7,8] needs special inertial sensors.
- It is suitable for positioning adopting a wide range of mobile devices, including both Android devices and IOS devices. While Wi-Fi-based positioning is only suitable for Android devices, because the API of IOS does not provide the Wi-Fi scanning results.
- The deployment cost for BLE beacons is low. Once the beacons are deployed, it can continuously work for a long duration (e.g., half a year) using their inner batteries, because the beacon nodes have low power consumption.

The BLE beacon-based positioning methods consist of mainly two types: range-based and fingerprinting-based. Range-based methods adopt a pre-defined radio frequency (RF) path-loss model to estimate the distance between the receivers (users) and the beacons. Assuming at least three received signal strength indication (RSSI) measurements are available, the user’s position can be solved by trilateration according to the distances estimated accordingly. The authors in [9] proposed three different methods for trilateration.

- The least square estimation (LSE) method [9]. By minimize the square sums of the distance errors, an optimal position can be found.
- The three-border method. By establishing the equations which represent the distance between the user and the beacons, the user’s position can be found by solving the equations.
- The centroid method. A polygon is firstly defined according to the vertexes defined by the intersecting points from the distance arcs. Then the centroid is regarded as the user’s position.

In [10], a more sophisticated adaptive propagation model is proposed, which models the parameters of the path-loss model with a particle filter. In [11], a combination of channel-separate polynomial regression model is adopted for estimating the distances between the receiver and the beacons, and the results show better accuracy than a simple propagation model.

The fingerprinting-based method consists of two phases: the offline phase and the online phase. In the offline phase, the fingerprints (a vector of RSSIs from different beacon nodes collected at a coordinate point) at different places are collected to create a reference fingerprint map (RFM). In the online phase, a fingerprint collected at an unknown place is compared with the fingerprints in the RFM, to solve for the position of the user. The online phase is rather simple and a commonly seen method for the phase is the k-nearest neighbor (kNN) method. However, as fingerprints needs to be collected at different places at the offline phase, the workload is huge. Moreover, the positions where the fingerprints are collected needs to be known, which is challenging. Normally, the positions need to be measured through dedicated surveying methods. For example, in [11], the fingerprints are collected manually at many fixed positions, then these fingerprints are interpolated according to a Gaussian process regression model to generate an RFM.

As mentioned, the range-based BLE beacon positioning methods need to know the positions of the beacons to start with. Similarly, a pre-requisite for fingerprinting-based methods is the RFM. Existing methods mainly focus on the positioning methods and assumes the beacon positions and the RFM is known before positioning. However, in practical applications, determining the beacon positions or fingerprinting collection positions, especially for the indoor environment with a relatively large area, is an energy intensive and error prone task.

**Figure 1:**
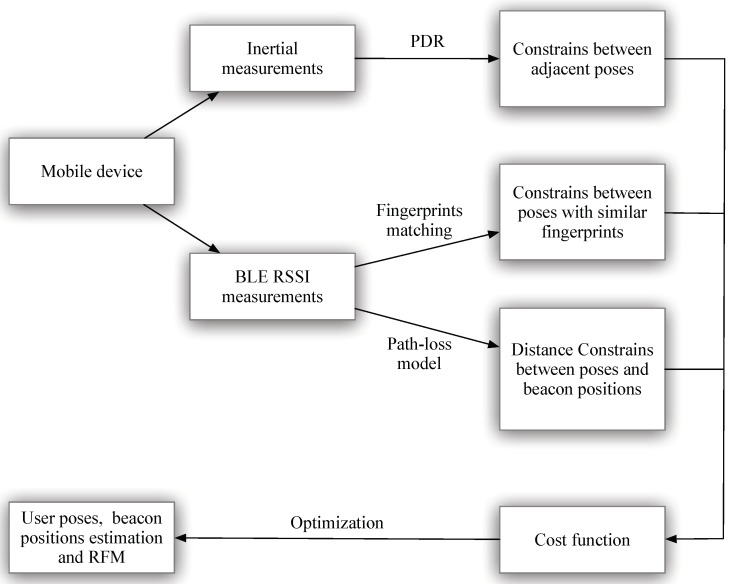
The overall data processing flow of the proposed method.

Unlike the mentioned methods, the proposed method in the paper provides an efficient way for estimating the pre-requisites for BLE-based positioning. The proposed method can estimate both the beacon positions and the RFM using graph-based optimization. The priority advantage of the proposed method is that it is free of any dedicated surveying process for the beacon positions and RFM. A user equipped with a BLE-enabled mobile device walks in the region. As shown in Figure 1, through the embedded sensors, the mobile device can collect inertial measurements and RSSI from BLE beacons. The method can fuse three types of information: the pedestrian dead reckoning (PDR) information, the fingerprint information, and the range information. More specifically, the inertial measurements are processed through the PDR method to generate the constraints at adjacent poses. In addition, the BLE fingerprints are adopted to generate constraints between poses (with similar fingerprints). The RSSIs are adopted to generate distance constraints between the poses and the beacon positions (according to a pre-defined path-loss model). The constraints are then adopted to form a cost function with a least square structure. By minimizing the cost function, the optimal user poses at different times and the beacon positions are estimated. In addition, the RFM can be generated through the pose estimations.

A few related works are given in Section 2 including the features of the BLE RSSI, PDR method and graph-based optimization. Section 3 is the method for forming the cost function and the optimization method. Experiments are carried out in Section 4 to validate the proposed method. The last section is the conclusion (Section 5).

## 2. Related Work

### 2.1. The BLE RSSI Features

BLE beacons operate in the same 2.4 GHz band as the Wi-Fi transceivers. Normally, in positioning, the beacons are in advertising mode, broadcasting short and flexible messages at flexible update rates [12,13]. Apple’s iBeacons are one type of such BLE beacons (supported by Android devices too), which also broadcast messages using the BLE standard [14]. The messages contain the ID of the beacons, so that the receiver can distinguish difference beacons. Different from the Wi-Fi APs, which broadcast messages at 20 MHz wide band, the BLE has 40 channels with width 2 MHz. Only three channels are adopted for broadcasting and each broadcasting is repeated for on the three channels. Due to the advertising scheme of the BLE beacons, the RSSI from these beacons have these features:

- The RSSI can change very dramatically with even a very small spatial change. As each of the broadcasting channels are narrow banded, the BLE signals have fast fading nature. Also, the indoor environment is very complex with many surfaces (e.g., walls, floors and so on), where the BLE signals can reflect. This can further cause multipath fades and add instability for the RSSI. In an experiment performed in [15], the noise and fading effect can even domain the RSSI with longer distance to the beacons.
- The RSSI can be reported for multiple times or not reported at all during a single scan. As the advertisement is repeated on three channels and if a scan is longer than a broadcasting interval, multiple reporting on the same beacon can be seen. Also, if the RSSI from all the three channels is below the environment noise due to fading effect, no reporting of the beacon is available for the scan. This can cause inconsistency for adjacent scans in time.

The mentioned features can cause huge noises in both RSSI (first feature) and beacon availability (second feature). To minimize the unfavorable effects, some smoothing processes should be added for the RSSI. In this paper, we adopt the median value of nearby RSSI (within a time window size of 1 s) as the current RSSI. This has been validated by an experiment in [13], which found out that 10 Hz beaconing and 1 s batch processing of median or mean values produced the best results.

### 2.2. Pedestrian Dead Reckoning

The PDR algorithm can update the pedestrian’s positions adopting the inertial measurements. Normally, the position update rate relies on the step rate of the pedestrian, i.e., the positions are updated each time a new step is taken by the pedestrian (in Equation (1)). (x,y) are the positions, the subscripts are the step indexes, *L* is the stride length estimation and θ is the orientation estimation.

(1) $$\begin{array}{c} x_{k}=x_{k-1}+L_{k}cos\left(\theta_{k}\right)\\ y_{k}=y_{k-1}+L_{k}sin\left(\theta_{k}\right) \end{array}$$

The details of the PDR algorithm differ with the body part where the inertial measurement unit (IMU) is mounted. If the IMU is mounted on the foot, the zero-velocity update (ZVU) method is adopted. In this method, a filtering process (assuming zero velocity) is added to the normal inertial calculation when the foot of the pedestrian touches the ground (zero-velocity phase) [16,17]. If the IMU is waist-mounted or hand held, no obvious zero-velocity phase can be found. Only the orientation is estimated through inertial calculation, while the stride occurrences and stride length are estimated through heuristic models [18]. In our implementation, as the inertial sensors in the mobile device is hand held, the second processing method of PDR is adopted. However, as the PDR algorithm relies heavily on heuristic assumptions, the accuracy is not satisfactory. Moreover, the insufficient accuracy of the gyroscopes can deteriorate orientation estimation. Even with magnetometer readings, the orientation estimation cannot be improved much, due to the many magnetic disturbances in the indoor environment [19]. Here, the PDR algorithm can only provide a rough constraint between pose estimation at adjacent time (here the PDR constraints denotes the stride length and orientation difference estimation between steps, which will be explained in the next section). Other types of constraints are needed to achieve pose estimations with reasonable accuracies.

### 2.3. Graph-Based Optimization

Graph-based optimization is currently widely adopted in robotics for solving the simultaneously localization and mapping (SLAM) problem [20]. It aims to estimate the poses of the robotics while generating a map of the environments (e.g., landmark maps, occupancy grid maps, feature maps and so on). The SLAM procedures consist of front-end tracking and back-end optimization.

- In the front-end tracking procedure, sensors on the robots are adopted to find constraints between the poses at different times. For example, features in adjacent image frames are adopted to find position and attitude constraints between poses (visual odometry) [21]. These constraints can generate a sequence of initial poses at different times (trajectory). However, this trajectory has accumulative error. If other types of sensor measurements (other than odometry observations) are available, more constraints can be formed, and the trajectory can be estimated more accurately using the back-end optimization.
- The back-end optimization procedure is generally establishing a cost function and then minimize it. The previously mentioned odometry observations and all other available observations are adopted to generate constraints between pose variables. Each constraint denotes a term in the cost function, which represents the errors between the sensor observation and the observation derived from pose variables. The cost function has a least square form which is in essence representing how the observations match the estimation. By finding the best match (minimizing the cost function), the optimal poses (trajectory) and maps can be found. The establishment of constraints and optimization process in our case is explained in detail in the next section.

**Figure 2:**
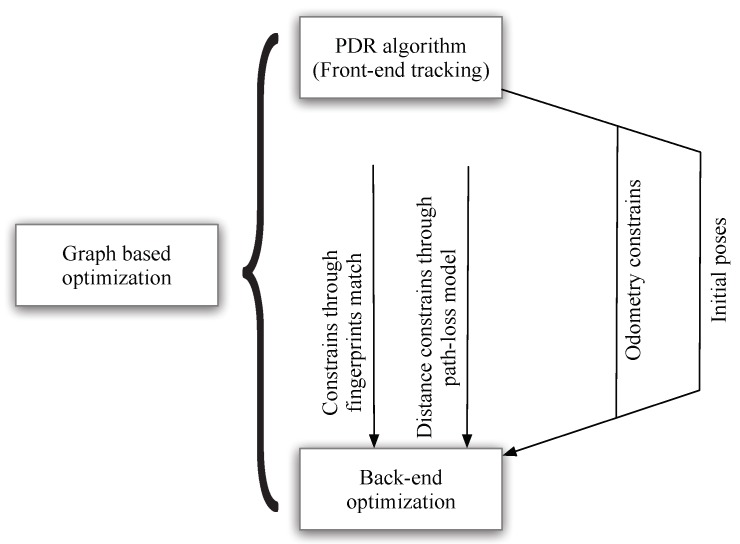
The graph SLAM procedures and its implementation in our method.

As shown in Figure 2, in our implementation, the front-end tracking procedure corresponds to generate initial and less accurate poses through the results of PDR algorithm. The back-end optimization procedure corresponds to finding the optimal poses and beacon positions from the cost function using three types of constraints: PDR constraints, constraints through fingerprints matching and distance constraints derived from the path-loss model. Noting that the initial poses correspond to the poses estimated solely from the PDR algorithm, and is adopted as initial values for the back-end optimization procedure. The front-end PDR algorithm is already well studied in a wide range of publications and considered trivial. We focus on adopting the constraints to build the back-end optimization procedure.

## 3. Method

The problem of user poses and beacon position estimation in our method is transformed to a least square optimization problem. In this section, a general least square optimization framework for user pose estimation is given at first. Then the framework is elaborated by adding least square terms according to the mentioned constraints to the cost function. After optimization using the Levenberg-Marquardt algorithm [22], the optimal user poses and beacon positions can be found.

### 3.1. Fundamentals of the Least Square Optimization Framework

The model for general pose estimation is formed at first. Assuming the noises are Gaussian, the maximum likelihood estimation of the poses is equivalent to optimize the formed cost function. Some fundamentals for the optimization process are given here.

#### 3.1.1. Formation of Least Squares

In a general pedestrian positioning problem, the poses of the pedestrian and the observations satisfy:

(2) $$\begin{array}{c} q_{t}=f\left(q_{t-1},s_{t}\right)+w_{t}\\ o_{t}=g\left(q_{t},\left(.\right)\right)+v_{t} \end{array}$$

Here, the pose at time *t* is denoted by $q_{t}$ and it is a three-element vector $\left[x_{t},y_{t},\theta_{t}\right]$. The observation at time *t* is denoted by $o_{t}$. $s_{t}$ is the from the PDR algorithm, which denotes the pose change between the ${t-1}^{th}$ pose and the $t^{th}$ pose. Here it is a two-element vector $\left[L_{t},\delta \theta_{t}\right]$ which is stride length estimation and heading change estimation respectively according to the PDR formation in Equation (1) (also denoted as odometry observation). $w_{t}$ and $v_{t}$ are Gaussian noise and is assumed to be independent at different times. The f(.) and g(.) are functions correspond to the pose propagation model and the observation model, respectively. In the propagation model, the pose at the next step $q_{t}$ is dependent on the previous pose $q_{t-1}$ and the pose change $s_{t}$ derived from the PDR algorithm. In the observation model, the current observation $o_{t}$ is dependent on the current pose $q_{t}$ and other variables (represented as (.) in Equation (2)). Noting that in real implementation, (.) can denotes another pose or other new variables need to be estimated. For generalization reasons, (.) is adopted instead.

In the formation, as the noises are assumed to be Gaussian, the maximum likelihood estimation of the poses is equivalent to finding the optimal poses which minimize the cost function with a least square form in Equation (3). The detailed proof of the equivalence can be found in [20].

(3) $$F\left(q\right)=\sum_{t}e_{t,f}W_{t}^{-1}e_{t,f}^{T}+\sum_{t}e_{t,g}V_{t}^{-1}e_{t,g}^{T}$$

where

(4) $$\begin{array}{c} e_{t,f}=q_{t}-f\left(q_{t-1},s_{t}\right)\\ e_{t,g}=o_{t}-g\left(q_{t},\left(.\right)\right) \end{array}$$

The error $e_{t,f}$ denotes the difference between the actual pose and the pose derived from the previous pose and the odometry observation from the PDR algorithm. The $e_{t,g}$ denotes the actual observation and the derived observation. The matrix W and V denote the noise variance of w and v respectively. We present the details of noise variances in the respective error terms later. From Equation (3), we can see that it can represent how the observations (including odometry observations) is consistent with the pose estimations. By minimizing F(q), the optimal pose estimations can be found which can best satisfy the observations.

**Figure 3:**
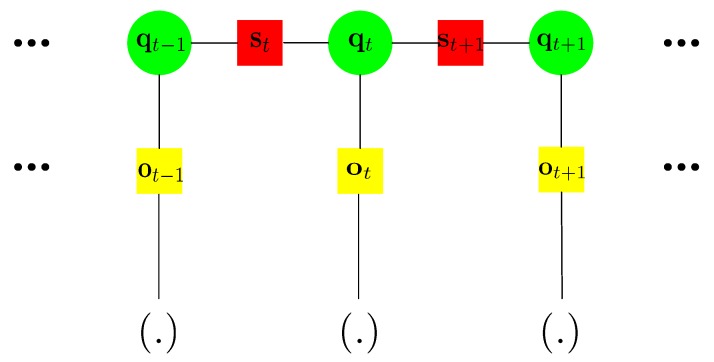
The factor graph representation of the cost function in Equation (3).

A factor graph can be adopted to better represent the cost function and relationships of the variables in it. In Figure 3, the circles denote the variables to be estimated (here the poses $q_{t}$). A square denotes a constraint existing between the variables they connect. Each square can correspond to a square term in Equation (3). Here are two types of constraints (squares). $s_{t}$ denotes the PDR derived odometry constraints between adjacent pose variables, which correspond to the term $e_{t,f}W_{t}^{-1}e_{t,f}^{T}$ in Equation (3). $o_{t}$ denotes the observation constraints between a pose variable and another variable (.), which correspond to the term $e_{t,g}V_{t}^{-1}e_{t,g}^{T}$ in Equation (3). (.) can be a pose variable or other type of variables need to be estimated. For BLE beacon-based graph in our method, (.) denotes other pose variables and the beacon position variables, which will be explained later. The factor graph can give a good visual representation of the cost functions and the variables need to be estimated. Therefore, the term graph optimization is adopted to represent the minimization process of the cost function.

#### 3.1.2. Levenberg-Marquardt Based Graph Optimization

The general square optimization (g2o) framework is proposed in [20] and is adopted in our method. Here we give a simple review of how the graph optimization problem is solved. We re-write the cost function in a more general form as:

(5) $$F\left(x\right)=\sum_{k\in C}\underset{F_{k}}{\underset{︸}{e_{k}\left(.\right)\Omega_{k}e_{k}{\left(.\right)}^{T}}}$$

where

- x is the set of many variables to be estimated, and it is $x={\left(x_{1}^{T},\ldots ,x_{n}^{T}\right)}^{T}$;
- C is the set for possible *k*, if we assume there are *n* variables in the factor graph, then it can be written as k=(1,…,n);
- $\Omega_{k}$ denotes the information matrix for the $k^{th}$ constraints (or square error terms). Here the information matrix corresponds to the inverse of the noise variance in Equation (3);
- $e_{k}\left(.\right)$ represents the errors from constraints. As the error is dependent on one or more variables, it can be written as:
  (6) $$e_{k}\left(.\right)=e_{k}\left(x_{k}\right)=e_{k}\left(x\right)$$

Then we adopt the Levenberg-Marquardt algorithm for finding the variable values which minimize the cost function iteratively. Assuming that in the previous iteration, the variable values can be written as $\overset{˘}{x}$. Here we consider the initial values of the variables is the value for the first iteration. Then we use the following steps for finding the variable values for the next iteration:

1. Expand the $e_{k}\left(x\right)$ using first-order Taylor expansion at the variable value $\overset{˘}{x}$ (value for the previous iteration):
  (7) $$e_{k}\left({\overset{˘}{x}}_{k}+\Delta \right)=e_{k}\left(\overset{˘}{x}+\Delta \right)≃e_{k}+J_{k}\Delta$$
  where the $J_{k}$ is the Jacobian matrix for the error function $e_{k}\left(x_{k}\right)$ at the value $\overset{˘}{x}$. $e_{k}$ is the value for the error function $e_{k}\left(x_{k}\right)$ at $\overset{˘}{x}$. Δ is a minor increment for the variable x. Then we substitute the single error function (Equation (7)) to $F_{k}$ in Equation (5) and we can get:
  (8) $$\begin{array}{c} F_{k}\left(\overset{˘}{x}+\Delta \right)\\ =e_{k}{\left(\overset{˘}{x}+\Delta \right)}^{T}\Omega_{k}e_{k}\left(\overset{˘}{x}+\Delta \right)\\ ≃{\left(e_{k}+J_{k}\Delta \right)}^{T}\Omega_{k}\left(e_{k}+J_{k}\Delta \right)\\ =\underset{c_{k}}{\underset{︸}{e_{k}\Omega_{k}e_{k}^{T}}}+2\underset{b_{k}}{\underset{︸}{e_{k}\Omega_{k}J_{k}}}\Delta +\Delta^{T}\underset{H_{k}}{\underset{︸}{J_{k}^{T}\Omega_{k}J_{k}}}\Delta \\ =c_{k}+2b_{k}\Delta +\Delta^{T}H_{k}\Delta \end{array}$$
  Then the overall error function (Equation (5)) with a minor increment can be written as:
  (9) $$\begin{array}{c} F\left(\overset{˘}{x}+\Delta \right)=\sum_{k\in C}F_{k}\left(\overset{˘}{x}+\Delta \right)\\ ≃\sum_{k\in C}\left(c_{k}+2b_{k}\Delta +\Delta^{T}H_{k}\Delta \right)\\ =c+2b\Delta +\Delta^{T}H\Delta \end{array}$$
  where
  (10) $$\begin{array}{c} c=\sum c_{k}\\ b=\sum b_{k}\\ H=\sum H_{k} \end{array}$$
2. Calculate the derivation of Equation (9) over the minor increment Δ and makes it equal to zero (get the minimized error). We can get
  (11) HΔ=-b
  Here H is the information matrix of the system. By solving the linear system in Equation (11), an increment can be acquired as $\Delta^{*}$, then we update the variable value for the current iteration:
  (12) $$\overset{˘}{x}=\overset{˘}{x}+\Delta^{*}$$
  The Gauss-Newton algorithm solves for the increment in Equation (11). For the Levenberg-Marquardt algorithm, a damping factor is added to Equation (11) to control the increment step size (more robust than the Gauss-Newton algorithm in general) and we can get
  (13) (H+λI)Δ=-b
  According to the algorithm, if $F\left(\overset{˘}{x}+\Delta \right)<F\left(\overset{˘}{x}\right)$, we decrease the damping factor λ, otherwise we increase it to control the step size. The detailed strategy for choosing the suitable λ is described in [22]. Here we just give a simple principle.
3. Continue step 1 and step 2 until the increment norm ∥Δ∥ is less than a pre-defined threshold. Then the current iteration of variable value $\overset{˘}{x}$ is considered optimal for the variables.

The dimension of H grows with the dimension of the variables x. However, the sparsity nature of the matrix H can be adopted to efficiently solve the linear system in Equation (13).

### 3.2. BLE Beacon-Based Graph Optimization

The graph optimization method for a general least square cost function is described in the previous subsection. Here we focus on forming the cost function for BLE beacon implementation using the constraints from PDR, beacon-based range constraints and fingerprint-based constraints.

#### 3.2.1. Cost function for BLE Beacon Implementation

**Figure 4:**
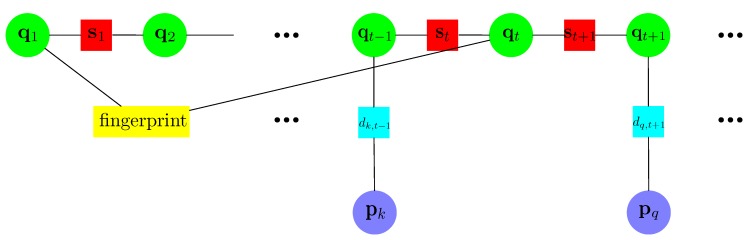
An illustration of the factor graph in BLE beacon implementation in our method.

Again, a factor graph is adopted to represent the cost function in Figure 4. Noting that the variable (.) in Figure 3 is specified here. There are three types of constraints here.

- The PDR constraints. Similar to Figure 3, the PDR constraints are between adjacent poses in time.
- The beacon position constraints. New variables representing beacon positions are added to the factor graph in Figure 4. For example, the distance $d_{k,t-1}$ between the pose variable $q_{t-1}$ and the $k^{th}$ beacon position variable $p_{k}$ is derived from the RSSI according to the path-loss model.
- The fingerprints matching constraints. These types of constraints are between two pose variables whose collected fingerprints are with vicinity. For example, Figure 4 shows that the fingerprint collected at $q_{1}$ and $q_{t}$ are with vicinity.

Based on the factor graph, we elaborate the square error terms of the cost function based on the different types of constraints.

#### 3.2.2. Error Terms for PDR-Based Constraints

As mentioned before, the pose variable $q_{t}$ here has three components ${\left[x_{t},y_{t},\theta_{t}\right]}^{T}$, which are the coordinate positions and the heading. From the PDR algorithm, the pose changes can be “observed” and can be written as

(14) $$s_{t}^{pdr}=\left[\begin{array}{c} L_{t}^{pdr}\\ \Delta \theta_{t}^{pdr} \end{array}\right]$$

where $L_{t}^{pdr}$ and $\Delta \theta_{t}^{pdr}$ are the “observed” stride length and heading change, respectively. As mentioned before, the error term for PDR-based constraints is dependent on adjacent pose variables $q_{t-1}$, $q_{t}$ and the “observed” stride length $L_{t}^{pdr}$ and heading change $\Delta \theta_{t}^{pdr}$ (components of $s_{t}^{pdr}$). We write it as

(15) $$e_{pdr}\left(q_{t-1},q_{t},s_{t}^{pdr}\right)=\left[\begin{array}{c} \sqrt{{\left(x_{t}^{q}-x_{t-1}^{q}\right)}^{2}+{\left(y_{t}^{q}-y_{t-1}^{q}\right)}^{2}}\\ \theta_{t}^{q}-\theta_{t-1}^{q} \end{array}\right]-\left[\begin{array}{c} L_{t}^{pdr}\\ \Delta \theta_{t}^{pdr} \end{array}\right]$$

where the $x_{t}^{q}$, $x_{t-1}^{q}$ and $\theta_{t}^{q}$ are the components taken from the pose variable $q_{t}$. Then the sum of the square error terms derived from PDR constraints can be written as

(16) $$F_{pdr}\left(.\right)=\sum_{t}e_{pdr}{\left(q_{t-1},q_{t},s_{t}^{pdr}\right)}^{T}\Omega_{pdr}e_{pdr}\left(q_{t-1},q_{t},s_{t}^{pdr}\right)$$

where the error term $e_{pdr}\left(q_{t-1},q_{t},s_{t}^{pdr}\right)$ is from Equation (15). The matrix $\Omega_{pdr}$ can be considered as a weighting matrix for the square error term. Normally, if the “observation” from the PDR results is accurate, the weighing matrix should be larger and thus making the square error term more significant in the overall cost function. In graph optimization, the weighting matrix is the inverse matrix of the noise variance. This also holds true for other types of error terms. The weighting matrix $\Omega_{pdr}$ is a 2×2 matrix and here it is:

(17) $$\Omega_{pdr}=\left[\begin{array}{cc} 1/0.5 & 0\\ 0 & 1/0.1 \end{array}\right]$$

here we consider the noise variance of the step length is 0.5 m${}^{2}$ and the noise variance of heading is 0.1 rad${}^{2}$ for each step in PDR.

To start the optimization, the initial values of the pose variable is considered the estimated poses from the PDR algorithm. If only one type of constraints (PDR-based constraints) exist in the cost function, the optimal poses should be the initial poses. In such a situation, the cost function is zero.

#### 3.2.3. Error Terms for Beacon Position Constraints

As mentioned before, the RSSI readings from BLE beacons have fast fading nature. Therefore, in our implementation, a pre-processing procedure is added to smooth the RSSI. Here the median value of the RSSI from a beacon within a time window (with duration 1 s) is taken as the current RSSI.

To estimate the distance from a beacon, a path-loss model is needed. Here we adopt the path-loss model in [23],

(18) $$\mathrm{RSSI}=-(10n\log_{10}d+A)$$

where parameter *A* is the absolute RSSI value represented by dBm at 1 m away from the beacon; *n* is a parameter related to the signal propagation environment and *d* is the distance from the beacon. In our implementation, we use the method proposed in [9] to estimate the parameters.

The path-loss model with pre-defined parameter in our implementation is

(19) $$\mathrm{RSSI}=-(10\times 2.5\log_{10}d+30)$$

An issue worth noting is that the model will be inaccurate when readings are higher than 0 dBm. However, we have checked our measurements and we have found out that none of RSSI readings are higher than 0 dBm. In our implementation, the BLE beacons adopted have the largest emitting power of 4 dBm and it is set to 4 dBm here. The reason maybe that, in the experiments, the beacons are installed on the walls and the person does not go too near to the walls.

**Figure 5:**
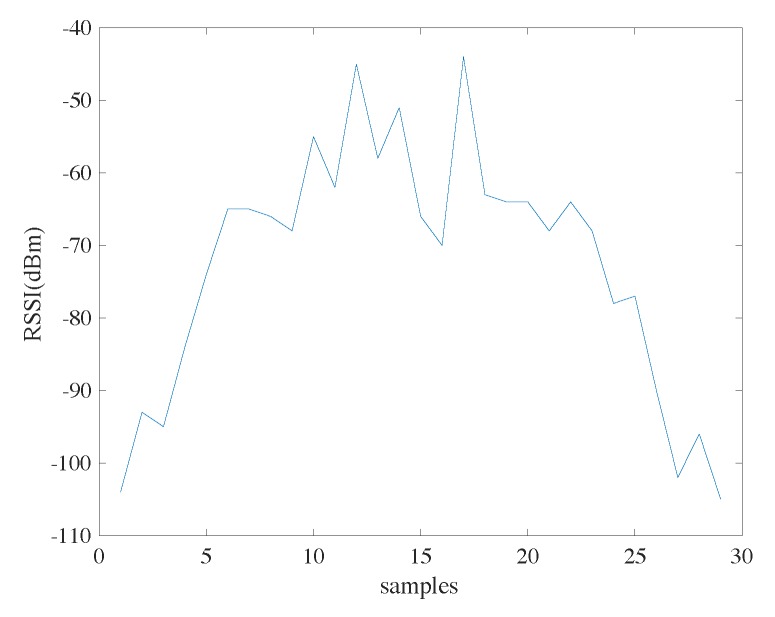
The RSSI readings when a user holding the receiver (phone) starts from a place far away from the beacon, walks near the beacon, and then leaves.

Here we only use the RSSI if it is larger than −100 dBm. A simple experiment is performed to explain the reasons. In an indoor corridor, the user holding the receiver starts from a place far away from the beacon, walks near the beacon, and then leaves. The RSSI readings are shown in Figure 5. We can see that the RSSI readings at lowest are a bit lower than −100 dBm (in this case, it is −105 dBm). This corresponds to the situation that the user is far away from the beacon, and the signal is buried in noise. In our implementation, we consider −100 dBm as the sensitivity of the receiver and RSSI readings smaller than −100 dBm (though seldomly seen) are discarded.

Assuming a RSSI reading for the $k^{th}$ beacon is available at the $t^{th}$ step. From the path-loss model, a distance $d_{k,t}$ can be derived from Equation (19). Then the error term derived from range-based constraint should be dependent on the pose variable $q_{t}$, the beacon position variable $p_{k}$ and the derived distance $d_{k,t}$. It can be written as

(20) $$e_{range}\left(q_{t},p_{k},d_{k,t}\right)=\sqrt{{\left(x_{t}^{q}-x_{k}^{p}\right)}^{2}+{\left(y_{t}^{q}-y_{k}^{p}\right)}^{2}}-d_{k,t}$$

where $x_{t}^{q}$ and $y_{t}^{q}$ are taken from the components in the pose variable $q_{t}$; $x_{t}^{p}$ and $y_{t}^{p}$ are taken from the beacon position variable $p_{k}$. Then the sum of square error terms derived from path-loss model and RSSI readings can be written as

(21) $$F_{range}\left(.\right)=\sum e_{range}{\left(q_{t},p_{k},d_{k,t}\right)}^{2}\Omega_{range}$$

where the sum is over all available ranges derived from RSSI readings. Again, $\Omega_{range}$ is the weight of the range-based square errors. $\Omega_{range}$ is a scaler 1/5 and we consider the noise variance in range estimation is 5 m${}^{2}$.

Another importance thing worth noting is that the initial values for the beacon position variables is needed to start the optimization. Here we consider no heuristic information about the beacons are available including the number of beacons and their positions. To start the optimization with initial guess of the beacon positions, we assume the initial beacon positions are at the poses from the PDR results when the beacons’ RSSI are available for the first time.

#### 3.2.4. Error Terms for Fingerprint Matching Constraints

For fingerprinting-based positioning using BLE beacon, an RFM should be established in the offline phase. The RFM consists of many fingerprints collected at different known positions. In our implementation, the user walks in the area collecting fingerprints. The optimal poses can provide the position estimations in the RFM.

**Figure 6:**
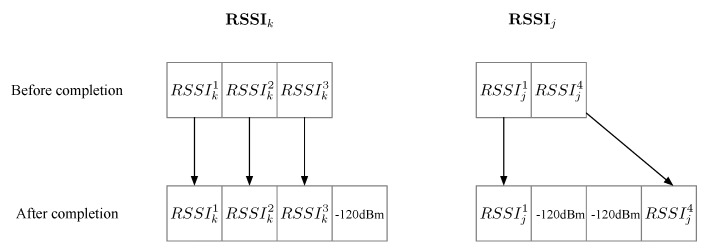
An example for the RSSI completion process before calculation of the RSSI distance.

In fingerprinting-based BLE positioning method, the distances between fingerprints can indicate the correlations for the positions. If two fingerprints are similar (with small distance), then the positions where the fingerprints are collected should be also with small distance (high correlation). We also use the mentioned feature for generating constraints through fingerprint matching. For such purposes, the distance metric is defined firstly according to [24],

(22) $$d\left({\mathrm{RSSI}}_{j},{\mathrm{RSSI}}_{k}\right)=\sqrt{\frac{\sum_{i=1}^{N}{\left({RSSI}_{j}^{\left(i\right)}-{RSSI}_{k}^{\left(i\right)}\right)}^{2}}{N}}$$

where the subscripts denote two different RSSI readings; ${RSSI}_{j}^{\left(i\right)}$ is the $i^{th}$ component in the vector ${\mathrm{RSSI}}_{j}$; *N* is the number of the beacons. Two RSSI vectors may have readings from different beacons. In this case, we add default readings to the RSSI vector such that both vectors have readings from the same beacon set. Here the beacon set denotes the union set of the two original beacon sets. An example is shown in Figure 6, where the original ${\mathrm{RSSI}}_{j}$ and ${\mathrm{RSSI}}_{k}$ have three and two components, respectively. After completion, they both become a 4-component vector with readings from the same beacons. The added default RSSI reading is set to −120 dBm in our implementation.

Assuming the two RSSI readings ${\mathrm{RSSI}}_{j}$ and ${\mathrm{RSSI}}_{k}$ correspond to the pose variables $q_{j}$ and $q_{k}$ respectively. The distance $d_{rssi}\left(j,k\right)$ between the RSSI readings can be calculated according to Equation (22). If the RSSI distance is less than a threshold $d_{rssi,thres}$ indicating high correlation on the corresponding positions, the error term of fingerprint matching-based constraint exists. The error term is

(23) $$e_{rssi}\left(q_{j},q_{k},{\mathrm{RSSI}}_{j},{\mathrm{RSSI}}_{k}\right)=\left\{\begin{array}{cc} 0, & d_{pos}\left(j,k\right)<d_{pos,min}\\ e_{ubound}, & d_{pos}\left(j,k\right)>d_{pos,max}\\ e_{ubound}\frac{d_{pos}\left(k,q\right)-d_{pos,min}}{d_{pos,max}-d_{pos,min}}, & \mathrm{otherwise} \end{array}\right.$$

where

- $d_{pos}\left(j,k\right)$ is the position distance calculated from the pose variables $q_{j}$ and $q_{k}$.
- $d_{pos,min}$ and $d_{pos,max}$ are the two position distance thresholds. If the position distance is less than $d_{pos,min}$, then the error should be 0, because we allow some position differences for high correlation poses. If the position distance is larger than $d_{pos,max}$, the error is considered to reach an upper bound $e_{ubound}$.
- If the position distance is between the two position thresholds, the error grows linearly with the position distance $d_{pos}\left(k,q\right)$.

The thresholds for fingerprint-based constraints in our implementation are in Table 1. Noting that the choice of these thresholds is similar to the implementation in [25], where the RSSI are from Wi-Fi APs. This set of parameters also works fine in our implementation. We will perform an exhaustive search for the optimal parameters in the future.

Then the sum of square error terms derived from fingerprint matching can be written as

(24) $$F_{finger}\left(.\right)=\sum e_{rssi}{\left(q_{j},q_{k},{\mathrm{RSSI}}_{j},{\mathrm{RSSI}}_{k}\right)}^{2}\Omega_{finger}$$

where the sum is over any two pose indexes whose fingerprint matches. Again, $\Omega_{finger}$ is the weight to control the error significance. $\Omega_{finger}$ is again a scaler 1/10 and we consider the noise in fingerprinting matching is 10 m${}^{2}$.

Then the overall cost function is the sum of the three types of constraints.

(25) $$F\left(.\right)=F_{pdr}\left(.\right)+F_{range}\left(.\right)+F_{finger}\left(.\right)$$

## 4. Experiment

### 4.1. Settings

In our experiments, we adopt the Google nexus 6p smart phone to collect the data. A custom Android application is designed for such purposes. In this application, the inertial data, including the acceleration, angular rate and the magnetometer readings are collected at a rate of 50 Hz. During the BLE scanning, the scan mode for the system is set to scan with low latency for continuous and fast scanning of the BLE beacons. In the Android system, this is done by setting the ScanSettings class to a constant as SCAN_MODE_LOW_LATENCY. The inertial data are processed in real time to estimate the coarse position changes for the person holding the phone in the hand adopting the PDR algorithm. The position changes along with the BLE scanning results (including the beacons’ names, mac addresses and RSSI values) are stored on the phone for offline processing. The BLE beacons adopted in our implementation are the Smart Beacons from the Bright Beacon company [26]. In our implementation, we set the broadcasting interval of the beacons to 300 ms and the emitting power to 4 dBm.

### 4.2. Accuracy for Positioning the Beacons

**Figure 7:**
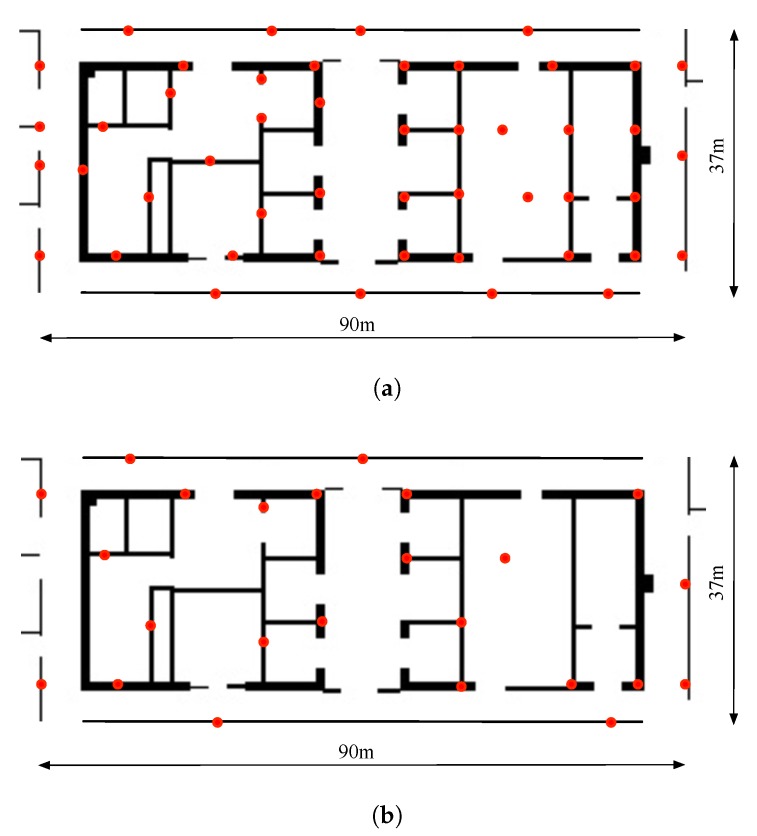
The positions of BLE beacons installed in the site (on the walls or on the furniture surfaces) (**a**) Densely deployed BLE beacons (**b**) Sparsely deployed beacons. In our implementation, we just ignore some of the RSSI readings to simulate the situation of sparsely deployed beacons

As shown in Figure 7, 48 BLE beacons are installed in an office site on the walls or on the surfaces of furniture. The beacons are installed approximate evenly covering the area. The size of the area is approximately 90 m × 37 m. Although there are 48 beacons installed, we manually take out half of the beacons (24 beacons) to see how the different number of beacons affect the performance of the proposed approach (in Figure 7). We refer to the different number of beacons as dense beacons and sparse beacons in the remaining of the paper. In this setting, the mean number of beacons over the minimum signal threshold for the dense beacon situation is about 6 and for the sparse case the number is about 3. The beacon positions are measured adopting a total station with sufficient accuracy and is regarded as ground truth positions. However, the ground truth positions of the beacons are only adopted for calculating the positioning errors of the beacons. During the optimization, we assume that the positions of the beacons are unknown and is estimated from the optimization process. We have labeled the beacons with different numbers and made a table of the numbers and the corresponding mac address of the beacons. In this way, we can identify the different beacons. A person holding the smart phone walked in the site for about 30 min with a total walking distance of about 1.6 km. The collected data is adopted for generating the aforementioned cost function. By minimizing the cost function, the beacon positions can be estimated. With the ground truth of the beacon positions, the position estimation errors can be acquired.

**Figure 8:**
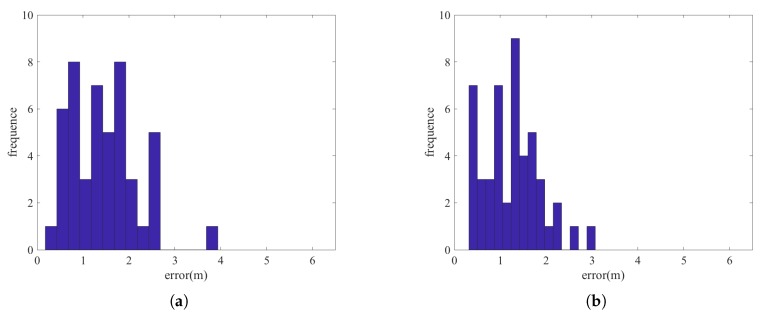
The histogram of beacon positions errors for dense beacons situation (**a**) Cost function with PDR constraint error terms and beacon position constraint error terms (**b**) Cost function with PDR constraint error terms, beacon position constraint error terms and fingerprint matching constraint error terms.

**Figure 9:**
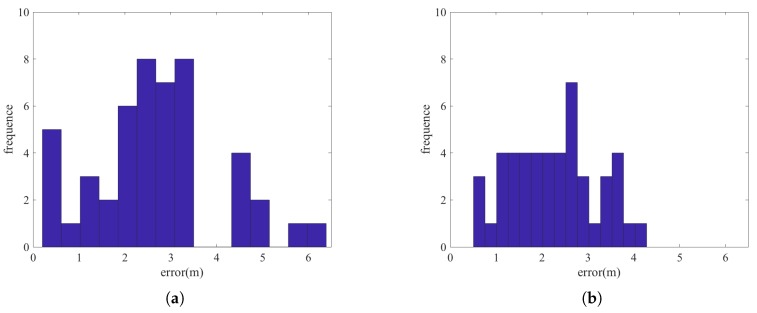
The histogram of beacon positions errors for sparse beacons situation (**a**) Cost function with PDR constraint error terms and beacon position constraint error terms (**b**) Cost function with PDR constraint error terms, beacon position constraint error terms and fingerprint matching constraint error terms.

Figure 8 and Figure 9 show the histograms of the errors for adopting the two types of error terms combination: PDR constraint terms + beacon position constraint terms and PDR constraint terms + beacon position constraint terms + fingerprint matching constraint terms. We can see that the incorporation of fingerprint matching error terms can improve the accuracy for positioning the beacons. Table 2 and Table 3 show some of the error statistics of the two combinations. For the proposed method (3 types of error terms combination), the mean positioning error of the beacons is only about 1.27 m in the dense beacon situation and 2.26 m for the sparse beacon situation. We show later that this level of beacon errors, especially for the dense beacon situation is not significant in deteriorating the performance of beacon-based positioning. However, for certain, this approach can estimate the beacon positions which is exempt from the heavy working load for carefully measure the beacon positions. In this experiment, it took 3 persons using nearly the whole day to measure the ground truth positions of the 48 beacons. Noting that here some large errors for beacon position estimations still exist. This may be due to the fact that the large noises in PDR results and the large noise in RSSI readings while the user is walking.

### 4.3. Accuracy for Beacon Based Positioning

As mentioned before, there are two types of beacon-based positioning method: range-based and fingerprinting-based. For range-based method, the positions of the beacons need to be known for positioning. For fingerprinting-based method, an RFM should be known for positioning. The proposed method in this paper gives an efficient way to estimate the beacon positions and to establish an RFM. To verify that the quality of the estimation for the beacon positions and the RFM is sufficient to support beacon-based positioning, another experiment is carried out. The person walked into the site again collecting BLE scanning RSSI readings. These RSSI readings are regarded as test data for showing the accuracy of beacon-based positioning. Here as the beacon-based positioning normally has many outliers, we adopt a simple extended Kalman filter (EKF)-based positioning method to fuse the beacon positioning results and the position update data from PDR. Noting that all the results including the results presented in Section 4.3.1 and Section 4.3.2 are results from the EKF. The adoption of the EKF for fusing PDR results and beacon positioning results is trivial and can be found in publications such as [11]. Therefore, how the EKF is implemented is not described in detail in the manuscript.

For measuring the ground truth of the persons, we have put some stickers to the ground and labeled the stickers with numbers on it to distinguish them. The stickers can be regarded as landmarks and the positions of landmarks are also measured through a total station prior to the positioning experiments. During the positioning experiments, the person can record the time and the landmark label during walking. This is done through pressing buttons on the APP designed for collecting data. In this way, the ground truth positions of the person when he/she walks to the landmarks are known. The positioning errors can then be determined through the differences between the estimated positions and the ground truth positions. In fact, we have set 50 landmarks roughly even distributed in the area. Since the positions of the landmarks should not affect the final results much, we did not show the positions of the landmarks.

#### 4.3.1. Accuracy for Range-Based Positioning

**Figure 10:**
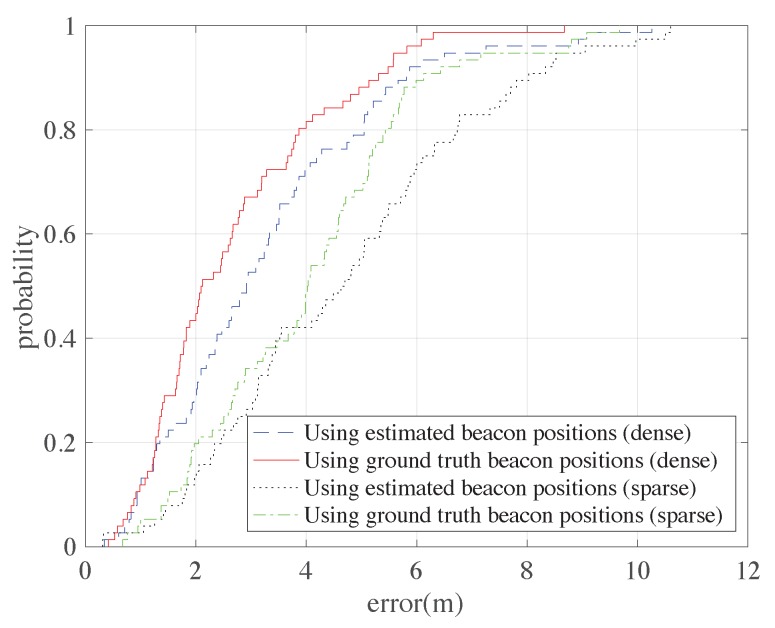
The CDFs of positioning errors using the ground truth beacons positions and the estimated beacon positions.

Here a classical type of range-based positioning is selected to test the positioning performance: the LSE method. By minimizing the square sums of the distance errors to the beacon positions, an optimal position can be found. Noting that here, the beacon positions should be known before positioning. Figure 10 shows the cumulative distribution functions (CDFs) of the positioning errors using ground truth beacon positions and estimated beacon positions (from the results in Section 4.2). Table 4 shows some of the error statistics of the two situations. It is not surprising that the positioning performance using the ground truth beacon positions is better than using the estimated beacon positions. However, the performance drop-down is not very significant with the mean error increase from 2.66 m to 3.25 m in the dense beacon situation and from 4.03 m to 4.69 m in the sparse beacon situation.

#### 4.3.2. Accuracy for Fingerprinting-Based Positioning

**Figure 11:**
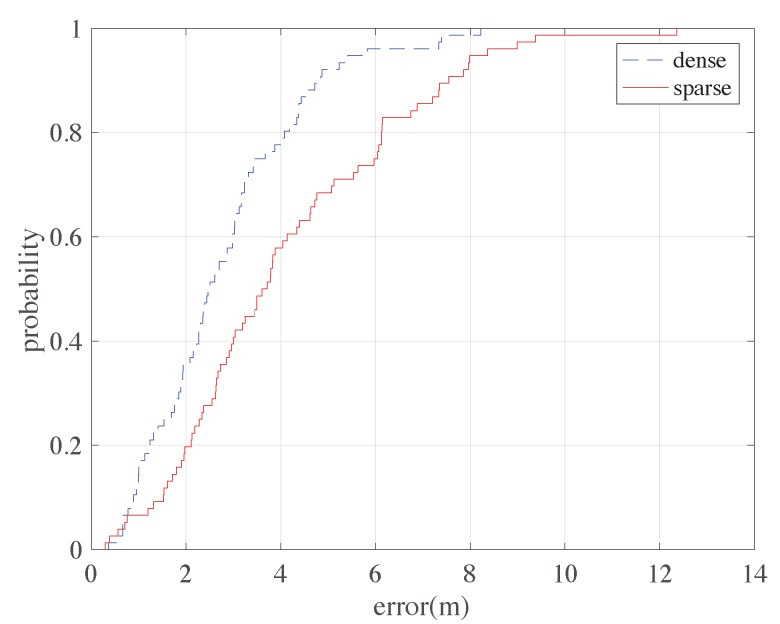
The CDF of positioning errors using the estimated RFM.

Our method can provide an RFM adopting the estimated positions of the person and the collected BLE RSSI readings. The RFM can be adopted for fingerprinting-based positioning. The positions of the collected fingerprints are firstly estimated adopting the proposed method. Then we partition the area to small grids with the size of 0.5 m × 0.5 m. The RSSI fingerprints at the centers of the grids are predicted through a simple Gaussian regression process adopting nearby fingerprints. These predicted fingerprints are then adopted to form the RFM in our implementation. Here the weighted kNN method is chosen for beacon-based positioning (in our case k = 3). The CDFs of positioning errors is shown in Figure 11. The mean error of fingerprinting-based positioning here is about 2.78 m for dense beacon situation and about 4.11 m for sparse beacon situation, which are considered sufficient for many indoor positioning applications. Noting that here the positioning performance is slightly better than the LSE method. This is consistent with the results in [11].

#### 4.3.3. Accuracy Comparisons for Fingerprinting Based Positioning and Range-Based Positioning

**Figure 12:**
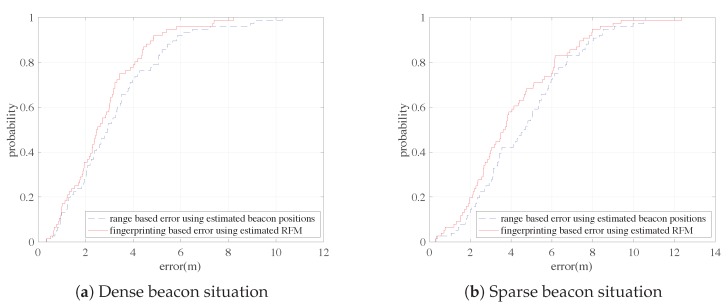
CDF comparison for range-based errors and fingerprinting-based errors under dense and sparse beacon situations using estimated beacon positions and RFM.

Fingerprinting-based positioning normally outperforms range-based positioning. This is also true in our experiments. The fingerprinting-based results and range-based results should be compared under the same condition. In fact, the fingerprinting-based results using the estimated Reference Fingerprint Map (RFM) should be compared to the range-based results using estimated beacon positions. We directly take the CDFs from our experiments and compare them in Figure 12a,b. It can be seen that the performance of fingerprinting-based positioning is better than range-based positioning under both dense and sparse beacon situations. Table 5 gives the mean error comparisons. We can see that the mean error of fingerprinting-based is 0.47 m less than range-based under dense beacon situation and 0.58 m less under sparse beacon situation. Noting that these results are directly taken from Section 4.3.1 and Section 4.3.2, and is presented differently here. The results have shown that the differences are not huge. This may due to the reason that range-based and fingerprint-based methods suffer or deteriorate differently on the inaccuracies of estimated beacons positions and RFM, respectively. This leads to another interesting topic which can be studied in the future.

#### 4.3.4. Accuracy Comparisons of the Proposed Method and Another Method

The proposed method provide an efficient way for estimating the beacon positions and the RFM without dedicated and time costly surveying process at the price of loss of positioning accuracy. However, considering the time and energy saved from dedicated surveying (it took three persons a whole day to measure the beacon positions using a total station in our situation), the method is meaningful.

A comparison to the method in [11] is presented here. The results comparisons are shown in Table 6. Noting that the accuracy of our method indeed is worse than that reported in [11]. However, for range-based case, our method is exempt of dedicated measuring of beacon positions, while in [11] the beacons are carefully measured with a range finder. For fingerprinting-based case, our method adopts an estimated RFM, while in [11] the RFM is constructed by exhaustive surveying 150 points in the site.

## 5. Conclusions

Most BLE beacon-based indoor positioning methods need some pre-requisites prior to positioning. For range-based methods, the beacons’ positions are needed. For fingerprinting-based methods, the RFM are needed. Surveying the beacon positions or the RFM is normally an energy intensive task. This paper proposes an easy way to estimate the pre-requisites for BLE beacon-based indoor positioning using graph optimization. On the hardware perspective, only the BLE beacons and a BLE-enabled mobile device is needed. The mean errors of the beacon positions are 1.27 m in dense beacon situation and 2.26 m in sparse beacon situation. Using the estimated pre-requisites, the BLE beacon positioning performance is considered enough for many indoor positioning applications, although it is worse than methods adopting accurate beacon positions and RFM.

In the future, we plan a deeper and more complete research into the proposed method on a wide range of affecting factors, including the number of beacons needed, the effect of walking persons in the environment, the effect of more sophisticated path-loss model, the upper bound error, and so on. 

## Figures and Tables

**Table 1:** The thresholds adopted for fingerprint-based constraints in our implementation.

Thresholds	Value
$d_{rssi,thres}$	8 dBm
$d_{pos,min}$	5 m
$d_{pos,max}$	20 m
$e_{ubound}$	1

**Table 2:** Some of the statistics of the beacon position errors for dense beacons situation.

Cost Function Error Terms	Mean Error (m)	Median Error (m)	Maximum Error (m)
PDR + beacon position	1.45	1.42	3.94
PDR + beacon position + fingerprint matching	1.27	1.28	3.07

**Table 3:** Some of the statistics of the beacon position errors for sparse beacons situation.

Cost Function Error Terms	Mean Error (m)	Median Error (m)	Maximum Error (m)
PDR + beacon position	2.68	2.65	6.39
PDR + beacon position + fingerprint matching	2.26	2.31	4.28

**Table 4:** Error statistics of range-based positioning using ground truth beacon positions and estimated beacon positions.

Beacon Positions	Mean Error (m)	50% Error(m)	70% Error (m)
ground truth positions (dense)	2.66	2.11	3.95
estimated positions (dense)	3.25	2.92	3.18
ground truth positions (sparse)	4.03	4.02	5.09
estimated positions (sparse)	4.69	4.66	5.90

**Table 5:** Mean error comparisons for range-based positioning and fingerprinting-based positioning using estimated beacon positions and RFM.

	Dense Beacon Situation (m)	Sparse Beacon Situation (m)
range-based	3.25	4.69
fingerprinting-based	2.78	4.11

**Table 6:** Mean positioning error comparisons of our method and the method reported in [11].

	Dense Beacon Situation (m)	Sparse Beacon Situation (m)
range-based (our method)	3.25	4.69
range-based (method in [11])	2.57	3.93
fingerprinting-based (our method)	2.78	4.11
fingerprinting-based (method in [11])	1.67	2.83

## Abbreviations

The following abbreviations are used in this manuscript:

BLE	Bluetooth low-energy
PbS	position-based service
RFM	reference fingerprinting map
RSSI	received signal strength indication
PDR	pedestrian dead reckoning
GNSS	global navigation satellite system
APs	access points
RF	radio frequency
LSE	least square estimation
kNN	k-nearest neighbor
IMUs	inertial measurement units
ZVU	zero-velocity update
RM	radio map
SLAM	simultaneous localization and mapping
EKF	extended Kalman filter
CDFs	cumulative distribution functions
